# Degradation of the LDL receptors by PCSK9 is not mediated by a secreted protein acted upon by PCSK9 extracellularly

**DOI:** 10.1186/1471-2121-8-9

**Published:** 2007-03-01

**Authors:** Øystein L Holla, Jamie Cameron, Knut Erik Berge, Trine Ranheim, Trond P Leren

**Affiliations:** 1 Medical Genetics Laboratory, Department of Medical Genetics, Rikshospitalet-Radiumhospitalet Medical Center, Oslo, Norway

## Abstract

**Background:**

Proprotein convertase subtilisin/kexin type 9 (PCSK9) post-transcriptionally degrades the low density lipoprotein receptors (LDLR). However, it is unknown whether PCSK9 acts directly on the LDLR or if PCSK9 activates another protein that in turn causes degradation of the LDLR.

**Results:**

We have transiently transfected HepG2 cells with wild-type and mutant D374Y-*PCSK9 *plasmids to study the effect of the conditioned medium on the LDLR of untransfected HepG2 cells. The ability of the conditioned medium to reduce the internalization of LDL was abolished by removal of recombinant PCSK9 from the conditioned medium by affinity chromatography. Thus, PCSK9 is the only factor in the conditioned medium able to mediate degradation of the LDLR. Moreover, fractionation of the conditioned medium by gel filtration showed that the ability of the fractions to reduce the internalization of LDL, closely paralleled the amount of D374Y-PCSK9 in the fractions. Incubation of a secreted, truncated LDLR without cytoplasmic and transmembrane domains, as well as membrane fractions from HepG2 cells, with conditioned medium containing PCSK9, did not reduce the amount of LDLR as determined by western blot analysis. Thus, the LDLR is not degraded by PCSK9 on the cell surface. The LDLR of HepG2 cells incubated with conditioned medium was protected from PCSK9-mediated degradation by the addition of nocodazole or ammonium chloride, but was not protected when the conditioned medium was made hypertonic. These findings indicate that the intracellular degradation of the LDLR involves intracellular transport along microtubules, an acidic intracellular compartment and that it occurs even when endocytosis through clathrin-coated pits has been blocked.

**Conclusion:**

Degradation of the LDLR by PCSK9 is not mediated by a secreted protein acted upon by PCSK9 extracellularly. Also the PCSK9-mediated degradation of the LDLR does not take place on the cell surface. Rather, the PCSK9-mediated degradation of the LDLR appears to take place intracellularly and occurs even when endocytosis through clathrin-coated pits is blocked by hypertonic medium.

## Background

Proprotein convertase subtilisin/kexin type 9 (PCSK9) is a proprotein convertase of the subtilase family [1,2]. It is synthesized as a soluble zymogen which undergoes autocatalytic intramolecular cleavage in the endoplasmic reticulum [1,2]. After cleavage, mature PCSK9 is efficiently secreted as a complex with its prosegment and can be found in the medium of cultured cells [1] and in human plasma[3,4]. By the use of subcellular fractionation, PCSK9 has been localized in endoplasmic reticulum and the intermediate vesicular compartment, but not in Golgi cisternae [5]. Even though the substrate for PCSK9 has not been identified, PCSK9 has been shown to play a role in cholesterol metabolism by regulating the number of cell surface low density lipoprotein receptors (LDLR) [6-9]. Because PCSK9 reduces the number of LDLR without reducing the amount of *LDLR *mRNA [6,7], degradation of LDLR by PCSK9 is apparently a post-transcriptional event.

Both the *LDLR *and the *PCSK9 *genes are transcriptionally regulated by sterol regulatory element-binding protein 2 [10,11]. Thus, both genes are upregulated when intracellular levels of cholesterol are low. A role for PCSK9 in the regulation of serum cholesterol levels in humans, is demonstrated by the findings that mutations in the *PCSK9 *gene have been associated both with hypo- [12-14] and hypercholesterolemia [15-20]. The mechanism by which some mutations cause hypocholesterolemia, and others cause hypercholesterolemia, is through reduced or increased LDLR-degrading activity, respectively [21]. A possible mechanism for the higher LDLR-degrading activity of the D374Y mutant PCSK9, is reduced inactivation by the proprotein convertases furin and/or PC5/6A [4]. For the S127R mutant, increased secretion of apolipoprotein B-containing lipoproteins may contribute to the hypercholesterolemia [22].

Degradation of the LDLR by PCSK9 is dependent on maintained catalytic activity of PCSK9 [7,8] and appears to take place in a post-Golgi compartment [7]. However, the exact location for the degradation has not been identified. We have previously shown that conditioned medium from HepG2 cells transiently transfected with a *PCSK9*-containing plasmid, reduces the amount of cell surface LDLR and internalization of LDL of untransfected HepG2 cells [21]. Thus, PCSK9 or a factor acted upon by PCSK9, is secreted from the transfected cells and degrades the LDLR either directly or indirectly. Even though an acidic compartment seems to be involved in degradation of LDLR by PCSK9 [7,23], studies of *ARH *knock-out mice suggest that internalization of LDLR is not required for the LDLR to be degraded by PCSK9 [8]. Thus, degradation may possibly take place on the cell membrane. However, no degradation products of the LDLR has been identified in culture medium of cells transfected with *PCSK9 *constructs. This indicates that PCSK9 may not simply cleave the LDLR on the cell surface or that the cleaved LDLR is rapidly degraded and has therefore escaped identification.

The ability of PCSK9 to degrade the LDLR is cell specific. Overexpression of PCSK9 reduces the number of LDLR in liver and kidney cells[8,23], but not in fibroblasts [8]. It is therefore possible that PCSK9 may require another cell-specific protein to exert its effect on the LDLR. Thus, the exact mechanism by which PCSK9 degrades the LDLR remains to be determined.

In this study we have examined the LDLR-degrading characteristics of conditioned medium from HepG2 cells transiently transfected with *PCSK9*-containing plasmids, to provide information about the mechanism by which PCSK9 degrades the LDLR. For these studies the *PCSK9 *cDNA construct containing mutation D374Y was primarily used because of its higher LDLR-degrading activity than the wild-type (WT) *PCSK9 *construct [4,21].

## Results

### Removal of PCSK9-His from conditioned medium restores internalization of LDL

**Figure 1:**
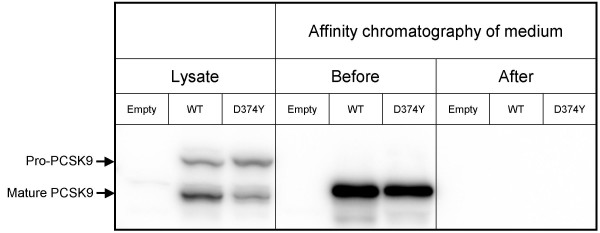
**Affinity chromatography of PCSK9-His from conditioned medium**. Conditioned media from HepG2 cells transiently transfected with empty plasmid, wild-type (WT)-*PCSK9*-His plasmid or D374Y-*PCSK9*-His plasmid were loaded on affinity columns containing a nickel-chelating resin. Conditioned medium before and after affinity chromatography was subjected to western blot analysis using an anti-PCSK9 antibody. Western blot analyses of cell lysates equivalent to 20 μg of protein per lane, were included to identify pro-PCSK9 and mature, cleaved PCSK9. Four independent experiments were performed from which one representative western blot is shown.

PCSK9 could either degrade the LDLR by a direct interaction, or PCSK9 could act on other proteins that in turn cause degradation of the LDLR. Studies were therefore performed to determine whether removal of PCSK9 from conditioned medium affected the ability of conditioned medum to reduce the internalization of LDL. For these studies, conditioned media were prepared from HepG2 cells transiently transfected with WT-*PCSK9*-His plasmid, D374Y-*PCSK9*-His plasmid or with empty plasmid. The conditioned media were subjected to affinity chromatography using columns containing a nickel-chelating resin that binds His-tagged fusion proteins. Western blot analysis showed that all WT-PCSK9-His and D374Y-PCSK9-His of the conditioned medium, had been removed from the conditioned medium by the affinity chromatography (Fig. 1). The slight upward shift of the band corresponding to the mature PCSK9 in the medium, as compared to the corresponding band in the lysate, is due to posttranslational modification of secreted PCSK9 [1].

**Figure 2:**
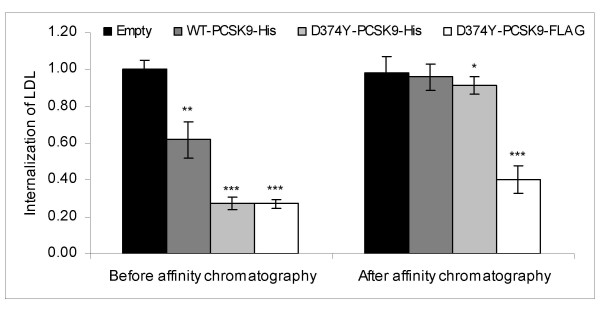
**Effect of conditioned medium depleted of PCSK9-His on the internalization of LDL**. His-tagged PCSK9 was cleared from conditioned medium of HepG2 cells transiently transfected with empty plasmid, wild-type (WT)-*PCSK9*-His plasmid or D374Y-*PCSK9*-His plasmid, by affinity chromatography. Conditioned medium from HepG2 cells transfected with D374Y-*PCSK9*-FLAG was also subjected to affinity chromatography as a control for a PCSK9 fusion protein which does not bind specifically to the nickel-chelating resin. The effect on internalization of LDL of the conditioned media before and after affinity chromatography, was studied by flow cytometry in untransfected HepG2 cells incubated with these media. Results represent mean (± SD) of four experiments. P-values are from comparisons between conditioned medium from transfections with empty plasmid on one hand and the PCSK9-containing media on the other hand (* p < 0.05, ** p < 0.01, *** p < 0.001).

The effect of removing PCSK9-His from the conditioned media on the internalization of LDL, was determined by flow cytometry. Conditioned medium containing WT-PCSK9-His or D374Y-PCSK9-His, reduced the internalization of LDL by 38 (± 10)% (p < 0.01) and 73 (± 3)% (p < 0.0001), respectively, when compared to conditioned medium obtained from HepG2 cells transfected with empty plasmid (Fig. 2). After removal of WT-PCSK9-His or D374Y-PCSK9-His from the conditioned medium by affinity chromatography, the internalization of LDL was significantly increased (p < 0.05 and p < 0.001, respectively). This post-affinity chromatographic increase in the internalization of LDL was significantly greater (p < 0.005) than the post-affinity chromatographic increase in the internalization of LDL caused by D374Y-*PCSK9*-FLAG which does not bind specifically to the nickel-chelating resin (Fig. 2). These findings indicate that PCSK9 is the only protein in the conditioned medium that causes degradation of the LDLR.

**Figure 3:**
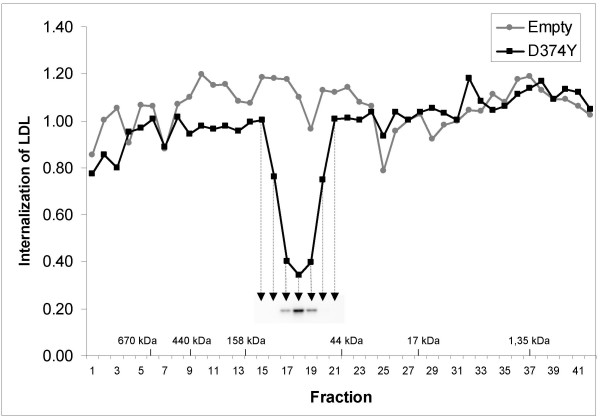
**Gel filtration of conditioned medium from HepG2 cells transiently transfected with D374Y-*PCSK9*-FLAG plasmid**. Conditioned medium from HepG2 cells transiently transfected with D374Y-*PCSK9*-FLAG plasmid or with empty plasmid were concentrated approximately 10 times and 1 ml was loaded onto a Superdex 200 gel filtration column. Fractions of 2 ml were collected. HepG2 cells were incubated with the different fractions to determine the effect on internalization of LDL by flow cytometry. Fractions containing molecular weight standards are indicated on the x-axis. Aliquotes of fractions 15–21 were also subjected to precipitation of proteins by trichloroacetic acid. The amount of mature D374Y-PCSK9-FLAG in the fractions was determined by western blot analysis using an anti-FLAG antibody as shown in the insert.

To further study whether the LDLR-degrading factor of the conditioned medium was PCSK9 itself and not a second protein acted upon by PCSK9, conditioned medium from HepG2 cells transiently transfected with D374Y-*PCSK9*-FLAG was fractionated by gel filtration on a Superdex 200 column. To determine the effect of the different fractions on the internalization of LDL, HepG2 cells were incubated with the individual fractions for 3 h at 37°C before the amount of LDL internalized was determined by flow cytometry. As can be seen from Fig. 3, cells incubated with fraction 18 had the lowest amount of LDL internalized. Slightly smaller reductions in the amount of LDL internalized were observed with fractions 17 and 19. Proteins in fraction 18 have a molecular hydrodynamic volume similar to that of proteins with a molecular weight of approximately 80 kDa. Western blot analysis of the fractions using an anti-FLAG antibody, showed that D374Y-PCSK9-FLAG were found in fractions 17–19 with the highest amount in fraction 18 (Fig. 3). Thus, the ability of the fractions to reduce the internalization of LDL, closely paralleled the amount of PCSK9 in the fraction. This finding therefore supports the notion that degradation of the LDLR by PCSK9, is caused by PCSK9 itself and not mediated by a second extracellular protein acted upon by PCSK9.

### PCSK9 does not degrade a secreted truncated LDLR

**Figure 4:**
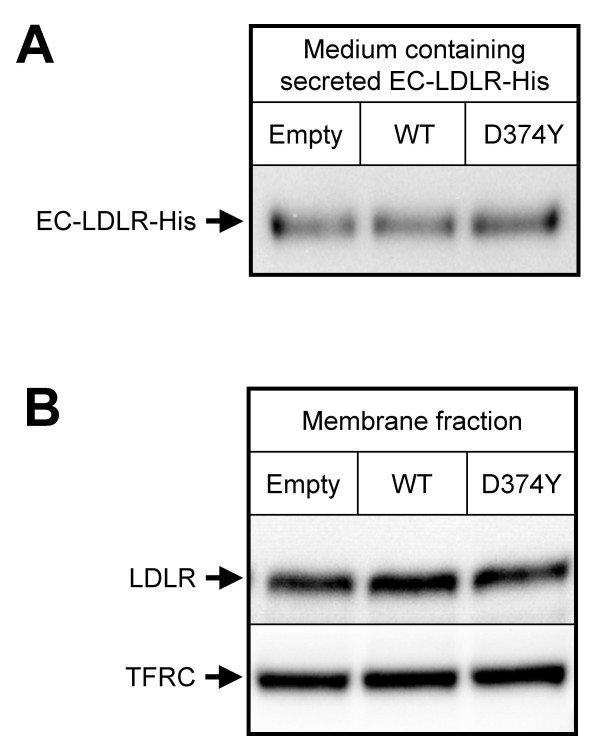
**Effect of conditioned medium containing PCSK9 on a truncated LDLR (EC-LDLR-His) and on the LDLR in membrane fractions**. **A) **Medium containing a secreted, truncated LDLR (EC-LDLR-His) missing the cytosolic and transmembrane domains of the LDLR was concentrated and incubated for 3 h at 37°C with conditioned media from HepG2 cells transiently transfected with empty plasmid, WT-*PCSK9*-His plasmid or the D374Y-*PCSK9*-His plasmid. **B) **HepG2 cell membrane fractions were isolated by ultracentrifugation and incubated for 3 h at 37°C with conditioned media from HepG2 cells transiently transfected with empty plasmid, WT-*PCSK9*-His plasmid or the D374Y-*PCSK9*-His plasmid. After incubations, both EC-LDLR-His and membrane fractions (15 μg per lane) were subjected to western blotting in order to determine the amout of LDLR. For the membrane fractions, the amount of transferrin receptor (TFRC) was used as control. Three independent experiments were preformed from which one representative western blot is shown.

To study whether WT-PCSK9 or D374Y-PCSK9 could degrade the LDLR independent of any intracellular compartments, a truncated LDLR (EC-LDLR-His) lacking the cytoplasmic and transmembrane domains of the LDLR was used. Upon synthesis, this truncated LDLR is secreted into the culture medium. Taking advantage of this, we incubated medium containing EC-LDLR-His with conditioned media containing either WT-PCSK9-His or D374Y-PCSK9-His. Conditioned medium from HepG2 cells transfected with empty-plasmid was used as a control. After incubation, the amount of EC-LDLR-His was determined by western blot analysis using an anti-LDLR antibody. As can be seen from Figure 4A, no significant difference in the amount of EC-LDLR-His was detected after incubation with conditioned medium containing WT-PCSK9-His or D374Y-PCSK9-His. This finding therefore suggests that PCSK9 does not degrade the LDLR by a direct action on the extracellular part of the LDLR. However, it is possible that degradation of the LDLR on the cell surface by PCSK9 could require the presence of other cell membrane proteins.

### PCSK9 in conditioned medium does not degrade the LDLR of membrane fractions

In order to determine whether PCSK9 could degrade the LDLR in the presence of other membrane proteins, membrane fractions from HepG2 cell were prepared by ultracentrifugation. The membrane fractions were incubated with conditioned medium from HepG2 cells transiently transfected with empty plasmid, WT-*PCSK9*-His plasmid or D374Y-*PCSK9*-His plasmid. Western blot analyses were performed to determine the amount of LDLR in the membrane fractions after incubation with the conditioned media. As can be seen from Figure 4B, the presence of PCSK9 in the conditioned media did not reduce the amount of LDLR in the membrane fractions. This finding indicates that even in the presence of other membrane proteins, PCSK9 does not degrade the LDLR on the cell surface. Thus, it seems that intact cells with intracellular compartments are required for the PCSK9-mediated degradation of the LDLR.

### PCSK9-mediated degradation of LDLR occurs independently of clathrin-coated pits

**Figure 5:**
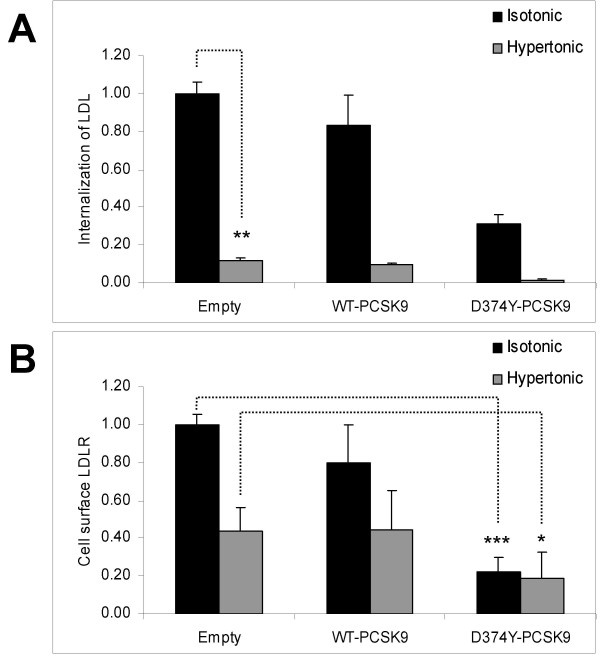
**Efect of hypertonic, conditioned medium on internalization of LDL and on the amount of cell surface LDLR**. HepG2 cells were transiently transfected with empty plasmid, WT-*PCSK9*-His plasmid or D374Y-*PCSK9*-His plasmid. Conditioned media from transfected cells were made hypertonic by addition of NaCl which increased the final concentration of NaCl by 150 mmol/l. The hypertonic medium was added to untransfected HepG2 cells for a 3 h incubation at 37°C. Isotonic, conditioned medium which had not been added NaCl was used as control. **A) **To measure internalization of LDL the cells were added fluorescently labelled DiD-LDL (10 μg/ml) and incubated for 2 h at 37°C. **B) **To measure the amounts of cell surface LDLR, the cells were labelled with anti-LDLR IgG-C7 antibody and counter-stained with Alexa Fluor^® ^488 goat anti-mouse IgG. Cell fluorescence was quantified by flow cytometry. Results represent mean (± SD) of three experiments (* p < 0.05, ** p < 0.01, *** p < 0.001).

To study whether endocytosis through clathrin-coated pits is required for the PCSK9-mediated intracellular degradation of the LDLR, conditioned medium was made hypertonic by adding 150 mmol/l of NaCl in order to rapidly and reversibly block clathrin-coated pit formation [24,25]. As a consequence of disruption of clathrin-coated pits, the LDLR are distributed over the entire cell surface [24]. In untransfected HepG2 cells incubated with hypertonic, conditioned medium from HepG2 cells transiently transfected with empty plasmid, the amount of LDL internalized was reduced to 12% (p < 0.01) as compared to regular, isotonic conditioned medium (Fig. 5A). Similar reduction in the internalization of LDL was also observed with hypertonic, conditioned medium from HepG2 cells transiently transfected with WT-*PCSK9*-FLAG or D374Y-*PCSK9*-FLAG plasmids (Fig. 5A). Thus, when the conditioned medium was made hypertonic, the LDLR-mediated uptake of LDL was severely reduced due to lack of LDLR-mediated endocytosis of LDL through clathrin-coated pits.

When the media were made hypertonic, the amount of cell surface LDLR was reduced by 57% (p < 0.05) and 45% (n.s.) in HepG2 cells incubated with conditioned medium from HepG2 cells transfected with empty plasmid or the WT-*PCSK9*-His plasmid, respectively (Fig. 5B). In HepG2 cells incubated with D374Y-PCSK9-His conditioned medium, no significant change in the amount of cell surface LDLR was observed in hypertonic medium. Moreover, the amount of cell surface LDLR in hypertonic D374Y-PCSK9-His medium remained significantly (p < 0.05) lower than the corresponding value obtained with hypertonic, conditioned medium from HepG2 cells transfected with empty plasmid (Fig. 5B). Thus, endocytosis through clathrin-coated pits does not seem to be a prerequisite for the intracellular degradation of the LDLR by PCSK9.

**Figure 6:**
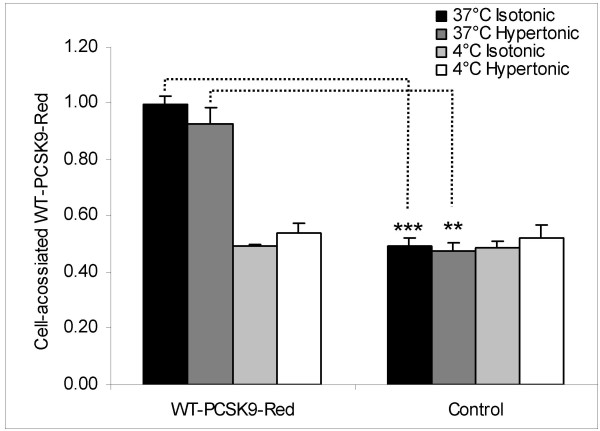
**Effect of hypertonic medium on internalization of WT-PCSK9-Red**. Conditioned medium from HepG2 cells transiently transfected with WT-*PCSK9*-Red plasmid was made hypertonic by addition of NaCl which increased the final concentration of NaCl by 150 mmol/l. The hypertonic, conditioned medium was added to untransfected HepG2 cells for 3 h incubations at 37°C or at 4°C. Isotonic, conditioned medium is conditioned medium without addition of NaCl. Amounts of cell-associated WT-PCSK9-Red were determined by flow cytometry. Control is medium without WT-PCSK9-Red. Results represent mean (± SD) of three experiments (** p < 0.01, *** p < 0.001).

To further study whether PCSK9 could enter HepG2 cells independently of clathrin-coated pits, the uptake of a red-tagged PCSK9 fusion protein (WT-PCSK9-Red) was studied by flow cytometry in HepG2 cells incubated with isotonic or hypertonic medium. The amount of WT-PCSK9-Red internalized is the difference between cell-associated WT-PCSK9-Red at 37°C and at 4°C. As can be seen from Fig. 6, HepG2 cells incubated with hypertonic medium containing WT-PCSK9-Red, was able to take up WT-PCSK9-Red although to a slightly lower extent (p < 0.05) than cells incubated with isotonic medium. Thus, PCSK9 is able to enter HepG2 cells independently of clathrin-coated pits.

### Nocodazole or ammonium chloride inhibits degradation of the LDLR

**Figure 7:**
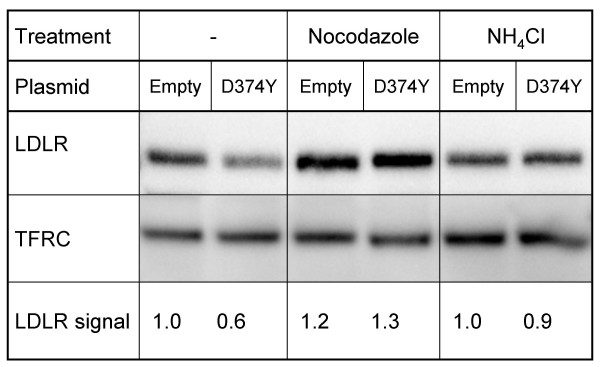
**Effect of nocodazole and ammonium chloride on PCSK9-mediated degradation of the LDLR**. HepG2 cells were cultured in media supplemented with nocodazole (20 μg/ml) or ammonium chloride (NH_4_Cl, 10 mM) for 30 min. The media were then replaced with conditioned media from HepG2 cells transiently transfected with D374Y-*PCSK9*-FLAG plasmid or with empty plasmid, already containing ammonium chloride or nocodazole, and the incubation was continued for 3 h. The conditioned media had also been added ammonium chloride or nocodazole. Cell membranes were isolated and membrane proteins equivalent to 10 μg were subjected to western blot analysis to determine the amount of LDLR. The amount of transferrin receptor (TFRC) was used as a control. Band intensities were quantified using a ChemiDoc XRS and Quantity One version 4.4.0 software. Results represent the mean (± SD) of four experiments, from which one representative western blot is shown.

Conditioned medium from HepG2 cells transiently transfected with the D374Y-*PCSK9*-FLAG plasmid, reduced the total amount of LDLR of untransfected HepG2 cells by 40% (p < 0.05) compared to conditioned medium from cells transfected with empty plasmid (Fig. 7). In contrast, conditioned medium from HepG2 cells transiently transfected with the D374Y-*PCSK9*-FLAG plasmid did not reduce the amount of cell membrane transferrin receptor (Fig. 7). Studies by others have suggested that degradation of the LDLR by PCSK9 takes place in an acidic intracellular compartment, possibly the lysosome [7,23]. Intracellular transport of an LDLR-containing endosome to the lysosomes requires intact microtubules that are disrupted by nocodazole [26]. To study whether nocodazole affected the LDLR-degrading activity of the conditioned medium, HepG2 cells were incubated for 3 h with conditioned medium from HepG2 cells transiently transfected with the D374Y-*PCSK9*-FLAG plasmid with or without addition of 20 μg/ml nocodazole. As can be seen in Fig. 7, nocodazole protected the LDLR from degradation by PCSK9. Nocodazole seemed to increase the amount of LDLR in cells incubated with conditioned medium from cells transfected with empty plasmid. Addition of ammonium chloride which increases the pH of acidic compartments also protected the LDLR from degradation by the conditioned medium (Fig. 7). The intracellular degradation of the LDLR by PCSK9 therefore seems to require transport along microtubules to an acidic compartment.

## Discussion

In this report we have performed studies of the LDLR-degrading activity of conditioned medium from HepG2 cells transiently transfected with *PCSK9*-containing plasmids. Such conditioned medium efficiently degrades the LDLR of untransfected cells [21]. The *PCSK9 *plasmid containing the mutation D374Y was primarily used as this mutant has a higher LDLR-degrading activity than WT-PCSK9 [4,21].

A key question regarding the mechanism by which PCSK9 degrades the LDLR, is whether PCSK9 acts directly upon the LDLR or whether it activates another protein which in turn causes degradation of the LDLR. To answer this question, the effect of conditioned medium on the LDLR was studied after D374Y-PCSK9-His had been removed by affinity chromatography. The results clearly showed that removal of WT-PCSK9-His or D374Y-PCSK9-His almost completely abolished the ability of conditioned medium to reduce the internalization of LDL. This indicates that the LDLR-degrading activity of PCSK9 is not mediated by another protein in the culture medium. The results of gel filtration of conditioned medium from HepG2 cells transiently transfected with D374Y-*PCSK9*-FLAG, showed that the LDLR-degrading activity of the fractions closely paralleled the amount of D374Y-PCSK9-FLAG in the fractions. This finding shows that PCSK9 purified by gel filtration degrades the LDLR when added back to cultured cells. Moreover, it suggests that PCSK9 degrades the LDLR in a dose-dependent manner. This finding also supports the notion that PCSK9 is the only extracellular protein involved in the degradation of LDLR. The possibility that the active form of PCSK9 was PCSK9 complexed with another soluble protein is unlikely as the proteins of the fraction obtained by gel filtration which had the highest LDLR-degrading activity, had a molecular hydrodynamic volume similar to that of proteins with a molecular weight of approximately 80 kDa. This is close to the molecular weight of approximately 75 kDa of mature PCSK9 complexed to its prosegment [1]. Together, these data indicate that PCSK9 is the only extracellular protein which causes degradation of the LDLR. These data also indicate that PCSK9 appears extracellularly as a monomer, and is not complexed to another protein. Consequently, the explanation for the cell-specific effect of PCSK9 on degradation of the LDLR, does not appear to be that PCSK9 requires a secreted cell-specific protein to degrade the LDLR. However, it is possible that PCSK9 may require the presence of cell-specific membrane-bound or cytosolic proteins to degrade the LDLR.

The failure of the secreted, truncated LDLR (EC-LDLR-His), which lacks the cytoplasmic and membrane-spanning domains, to be degraded when incubated with conditioned medium, indicates that PCSK9 does not degrade the LDLR directly by acting on the extracellular part of the LDLR. It is possible that degradation of the LDLR on the cell surface, could require a cell membrane protein not present in the pure preparation of the truncated EC-LDLR-His. However, incubation of membrane fractions with conditioned media did not lead to degradation of the LDLR of the membrane fractions. Thus, even in the presence of other membrane proteins, PCSK9 does not degrade the cell surface LDLR. It therefore seems that PCSK9 and/or the LDLR need to be internalized in order for PCSK9 to degrade the LDLR.

Our finding that D374Y-PCSK9-His was able to reduce the number of cell surface LDLR even when clathrin-coated pits were blocked by hypertonic medium, suggests that the intracellular degradation of the LDLR by PCSK9 does not involve endocytosis through clathrin-coated pits. This finding is therefore in agreement with the finding that functional ARH is not required for PCSK9 to degrade the LDLR in mice overexpressing PCSK9 [8]. However, these findings are at variance with the finding of Lagace et al. [27], which was published during review of our paper, who found that functional ARH is required for PCSK9 to degrade the LDLR when added exogenously to primary hepatocytes. The explanation for these conflicting data could be that compensatory clathrin-independent endocytotic pathways may become activated when the clathrin-coated pits are artificially blocked or when PCSK9 is overexpressed. The notion that the intracellular degradation of the LDLR may be independent of how the LDLR and PCSK9 enter the cells, is supported by the findings in HEp-2 cells where molecules endocytosed through clathrin-independent mechanisms were delivered to endosomes containing cargo which had been endocytosed through clathrin-coated pits [28]. Even though our studies show that clathrin-independent endocytosis is able to mediate the degradation of the LDLR by PCSK9, it is still possible that clathrin-dependent endocytosis may be the predominant pathway by which LDLR and PCSK9 enter the cells in vivo.

Both we, in this study, and others [7,23] have found that addition of ammonium chloride protects the LDLR from PCSK9-mediated degradation. This suggests that an acidic compartment is involved in the degradation of the LDLR. Together with our finding that nocodazole also protects the LDLR from degradation, transport of endosomes containing LDLR along microtubules to an acidic compartment seems to be involved in the degradation of LDLR by PCSK9. This notion is supported by the findings of Benjannet et al. [4] who have found present PCSK9 in both early and late endosomes. It is also supported by the recent data of Lagace et al. [27] who found that PCSK9 and LDLR associate and can be found in late endocytic compartments. They also found that administration of purified PCSK9 to the medium of HepG2 cells, reduced the number of cell surface LDLR in a dose- and time-dependent manner. Thus, it may seem that PCSK9 has to be secreted and taken up by the cells in order to shuttle the LDLR from recycling endosomes to a late endosomes and eventually to lysosomes. However, more studies are needed to determine the exact mechanisms by which PCSK9 degrades the LDLR intracellularly.

## Conclusion

Degradation of the LDLR by PCSK9 is not mediated by a secreted protein acted upon by PCSK9 extracellularly. Also the PCSK9-mediated degradation of the LDLR does not take place on the cell surface. Rather, the PCSK9-mediated degradation of the LDLR appears to take place intracellularly and occurs even when endocytosis through clathrin-coated pits is blocked by hypertonic medium.

## Methods

### Mutagenesis of PCSK9

D374Y-*PCSK9*-FLAG plasmid containing mutation D374Y was made by site-directed mutagenesis of the WT *PCSK9 *plasmid (pCMV-*PCSK9*-FLAG) which contains the sequence for the FLAG epitope tag fused to the 3' end of the PCSK9 coding sequence, as previously described [21]. To create the D374Y-*PCSK9*-His plasmid, D374Y-*PCSK9 *was first amplified using the D374Y-*PCSK9*-FLAG plasmid as a template and the 5' primer: 5'-CACCATGGGCACCGTCAGCTCCAG-3' and 3' primer: 5'-CTGGAGCTCCTGGGAGGCCTGCGC-3'. The PCR product was cloned into pcDNA3.1D/V5-His-TOPO with pcDNA3.1 Directional TOPO Expression Kit (Invitrogen, Carlsbad, CA). The resulting fusion protein (D374Y-PCSK9-His) contains D374Y-PCSK9 fused to a 47 amino acid peptide containing the V5 and His epitope tags. The WT-PCSK9-His plasmid was created by site-directed mutagenesis of the D374Y-*PCSK9*-His plasmid using the 5' primer: 5'-CATTGGTGCCTCCAGCGACTGCAGCACCTGC-3' and 3' primer: 5'-GCAGGTGCTGCAGTCGCTGGAGGCACCAATG-3'. An expression vector that encodes WT- PCSK9 followed by a red fluorescent protein was constructed as follows. The WT-*PCSK9*-FLAG plasmid was used as template for amplification of WT-*PCSK9 *using the primers: 5' primer; 5'-CCCTCGAGATGGGCACCGTCAGCTCCAGGCGG-3', 3' primer; 5'-ATCCCGGGCCTGGAGCTCCTGGGAGGCCTGCGCCA-3'. The 5' primer contained a XhoI restriction site, and the 24 first nucleotide of the translated part of the *PCSK9 *gene. The 3' primer contained a SmaI site and the last 27 nucleotides of the translated part of the *PCSK9 *gene. The stop codon was not included in the 3'-primer. The amplified PCR product was digested with XhoI and SmaI (New England Biolabs Inc., Ipswich, MA). The digested products were separated by agarose gel electrophoresis. The relevant band was excised and purified prior ligation into a similarly digested pDsRed-Express-N1 plasmid (Clontech Laboratories, Inc., Palo Alto, CA) to generate WT-*PCSK9*-Red. The integrity of the plasmids was confirmed by DNA sequencing. An empty plasmid, pcDNA3.1/c-myc-His (Invitrogen), was used as a negative control in transfection experiments.

### Production of conditioned medium from transiently transfected HepG2 cells

HepG2 cells (The European Collection of Cultured Cells, Wiltshire, UK) were cultured in Modified Eagle's medium (MEM) (Gibco, Carlsbad, CA), containing penicillin (50 U/ml), streptomycin (50 μg/ml), L-glutamine (2 mM) and 10% fetal calf serum (Invitrogen) in a humidified atmosphere (37°C, 5% CO_2_). For transient transfection experiments, HepG2 cells were seeded in 75 cm^2 ^collagen-coated flasks (BD Biosciences, San Diego, CA) and grown to 80% confluency. The cells were transiently transfected with *PCSK9*-containing plasmids or with empty plasmid using Fugene-6 Reagent (Roche Diagnostics GmbH, Mannheim, Germany) according to the manufacturer's instructions. A ratio of 6:1 reagent to plasmid DNA was used during transfections. Transfection efficiencies were in the range of 10–20%. 24 h after transfection, the transfection solution was removed and the cells were washed once with phosphate-buffered saline (PBS) before addition of 15 ml serum-free OptiMEM medium (Gibco). After a further 24 h incubation, the medium was removed and centrifuged for 5 min at 3000 rpm. This medium is referred to as conditioned medium. Constructs with a His sequence tag were used to generate PCSK9-His fusion proteins in order to enable immobilization of PCSK9-His on a nickel-chelating resin. Conditioned media from cells transfected with *PCSK9*-His constructs were also used for incubations with membrane fractions and with a preparation of truncated LDLR lacking the cytoplasmic and transmembrane domains as well as the experiments involving hypertonic media. For the other experiments, *PCSK9 *constructs with a FLAG sequence tag were used. The LDLR-degrading activity of conditioned medium from HepG2 cells transiently transfected with D374Y-*PCSK9*-FLAG plasmid, was maintained for at least two weeks when stored at 4°C (data not shown).

### Western blot analysis

Cells were lysed in a buffer containing 1% Triton X-100, 100 mM NaCl, 10 mM EDTA, 20 mM Tris-HCl (pH 7.5). Cell lysates equivalent to 20 μg protein, or membrane fractions equivalent to 10 or 15 μg were separated by gel electrophoresis using a 4–20% Tris-HCl Criterion Precast Gel (Bio-Rad, Hercules, CA). The proteins were then electrophoretically transferred to an Immun-Blot PVDF Membrane for Protein Blotting (Bio-Rad). Non-specific binding sites were blocked in 5% Blotting Grade Blocker Non-Fat Dry Milk (Bio-Rad) for 1 h or overnight, and the membrane was immunostained for 1 h. The antibodies used were rabbit IgG anti-LDLR (1:1000, Progen Biotechnik GmbH, Heidelberg, Germany), rabbit IgG anti-PCSK9 (1:200, Cayman Chemical Company, Ann Arbor, MI), mouse anti-human transferrin receptor (1:1000, Zymed, San Fransisco, CA), mouse IgG anti-human CD71 (1:1000, Nordic Biosite, Täby, Sweden) and mouse anti-FLAG M2 monoclonal antibody (1:3000, Sigma-Aldrich Corp., St. Louis, MO). After incubation with the primary antibody the membranes were washed twice in Tris-buffered saline (TBS) containing 0.1% Tween-20 (Sigma-Aldrich Corp.) and incubated for 1 h with sheep anti-rabbit IgG or sheep anti-mouse IgG (Amersham Biosciences, Little Calfont, UK), both conjugated with horseradish peroxidase. After two more washing steps with TBS containing 0.1% Tween-20, the bands were visualized with SuperSignal West Dura Extended Duration Substrate (Pierce Biotechnology, Rockford, IL) and chemiluminescence was detected on a ChemiDoc XRS (Bio-Rad). Quantity One version 4.4.0 software (Bio-Rad) was used for quantification of band intensities.

### Flow cytometric analysis of internalization of LDL and cell surface LDLR

A FACS Canto flow cytometer (BD Biosciences) was used to determine the amounts of LDL internalized by cultured cells and the amounts of cell surface LDLR, as previously described [21]. Briefly, LDL was fluorescently labelled with 1,1'-dioctadecyl-3,3,3',3'-tetramethylindodicarbocyanine perchlorate (DiD) (Molecular Probes, Eugene, OR), added to the cells (final conc. 10 μg/ml) and incubated for 2 h at 37°C. Cell surface LDLR were labelled with anti-LDLR IgG-C7 mouse monoclonal antibody (1:20, Progen Biotechnik) and counter-stained with Alexa Fluor^® ^488 goat anti-mouse IgG (H+L) (1:400, Molecular Probes). The amount of LDL internalized and cell surface LDLR were then determined by flow cytometry. LDLR levels were corrected for unspecific staining by labelling the cells with secondary antibody only.

### Gel filtration of conditioned medium

Conditioned medium was concentrated approximately 10 times using BJP concentrators (Pro-Chem Inc., Littleton, MA) and 1 ml was loaded on a Superdex 200 gel filtration column (Pharmacia Fine Chemicals, Uppsala, Sweden). PBS was used for elution at a flow rate of 1 ml/min and fractions of 2 ml were collected. Gel Filtration Standard (Bio-Rad) containing five different proteins with molecular weights ranging from 1.35 to 670 kDa was used together with ferritin (440 kDa) for size determination. To study the ability of the fractions to reduce the internalization of LDL, HepG2 cells were incubated with the different fractions for 3 h at 37°C. Fluorescently labelled LDL was then added and the cells were incubated for another 2 h at 37°C. The amounts of LDL internalized were determined by flow cytometry.

### Preparation of a truncated LDLR lacking the cytoplasmic and membrane-spanning domains

An *LDLR *construct encoding the 21 amino acids of the signal peptide, the 767 amino acids extracellular domain and the first amino acid of the membrane-spanning domain, was constructed by mutagenesis of the plasmid pcDNA4-*LDLR*-EYFP [29] which contains the coding sequence of the *LDLR*. This construct encodes the extracellular (EC) part of the LDLR. The primers used to amplify the truncated LDLR were: 5' primer: 5'-CTTGGTACCAGCATGGGGCCCTGGGGCTGGAAATTGC-3', 3' primer: 5'-CGAGCGGCCGCAGCCCTCACGCTACTGGGCTTCTTC. The 5' primer contained a KpnI restriction site, a Kozak sequence and the first 25 nucleotides of the translated part of the *LDLR *cDNA. The 3' primer contained a NotI site and nucleotides 2343–2467 of the *LDLR *cDNA. The amplified PCR product was TA-cloned into the pCR^® ^2.1-TOPO vector of the TOPO TA cloning kit (Invitrogen) according to the instructions by the manufacturer. The resulting pCR^® ^2.1-EC-*LDLR *plasmid was digested with KpnI and NotI (new England Bioloabs, Inc.), and the fragments were separated by agarose gel electrophoresis. The band corresponding to EC-LDLR was cut out of the gel and purified by QIAquick gel extraction kit (Qiagen, Hilden, Germany) prior to ligation into a similarly digested pcDNA3.1/c-myc-His plasmid (Invitrogen) to generate pcDNA3.1-EC-*LDLR*-His. This plasmid contains the truncated *LDLR *cDNA fused to the c-myc and His sequence tags. The integrity of the plasmid was verified by DNA sequencing. The truncated LDLR (EC-LDLR-His) without the cytoplasmic and membrane-spanning domains, was prepared from media of HepG2 cells transiently transfected with the pcDNA3.1-EC-*LDLR*-His plasmid. 5 ml of medium containing secreted EC-LDLR-His was concentrated to 200 μl using BJP protein concentators (Pro-Chem Inc.). 10 μl of this concentrated medium was then added to 50 μl conditioned medium containing either WT-PCSK9-His or D374Y-PCSK9-His. Conditioned medium from HepG2 cells transfected with empty plasmid was used as a control. After incubation for 3 h at 37°C, 20 μl was subjected to western blot analysis in order to quantify the amount of EC-LDLR-His.

### Cell membrane preparations

HepG2 cells were cultured in serum-free OptiMEM medium overnight and harvested by scraping. After sonication, the cells were centrifuged at 500 g for 10 min. The supernatant was removed and centrifuged at 100 000 g for 60 min in a Beckman Coulter Optima LE-80K ultracentrifuge (Beckman Coulter, Fullerton, CA) using an ultrarotor 70TI (Beckman Coulter). The pellet containing the cell membranes was dissolved in PBS. 25 μl of cell membranes was incubated with 25 μl conditioned medium from HepG2 cells transiently transfected with empty plasmid, WT-*PCSK9*-His plasmid or D374Y-*PCSK9*-His plasmid.

### Affinity chromatography of His-tagged fusion proteins

WT-PCSK9-His or D374Y-PCSK9-His secreted from HepG2 cells transiently transfected with WT-*PCSK9*-His or D374Y-*PCSK9*-His plasmids were isolated from 2 ml of culture medium added 500 μl 5× Native Purification Buffer with imidazole (final conc. 30 mM) using the ProBond Purification System (Invitrogen) according to the manufacturer's instructions. The Resin-Purification Column contains Nickel-Chelating Resin which selectively binds His-tagged recombinant fusion proteins. The cleared medium was sequentially dialyzed against PBS and OptiMEM serum-free medium at 4°C using a Slide-A-Lyzer Dialysis Cassette (10 000 MWCO, Pierce Biotechnology).

### LDLR in hypertonic medium

Hypertonic medium was prepared by a slight modification of the method described by Krämer-Guth et al. [25]. 30 μl of 5.0 M NaCl was added per ml of OptiMEM medium to increase he final concentration of NaCl by 150 mmol/l. The hypertonic medium was added to untransfected HepG2 cells for a 30 min preincubation at 37°C. The hypertonic medium was replaced with conditioned medium from transiently transfected HepG2 cells which had been made hypertonic after it had been removed from the transfected cells. The amount of cell surface LDLR in untransfected HepG2 incubated with hypertonic, conditioned medium, was determined by flow cytometry as previously described.

### Degradation of the LDLR in the presence of nocodazole or ammonium chloride

HepG2 cells were seeded in 25 cm^2 ^flasks (10^6 ^cells/flask) and cultured overnight. Nocodazole (Sigma-Aldrich Corp., final conc. 20 μg/ml) or ammonium chloride (Sigma-Aldrich Corp., final conc. 10 mM) was added to the cells and incubated for 30 min at 37°C. Medium was then replaced with conditioned medium containing nocodazole or ammonium chloride (final conc. 20 μg/ml and 10 mM, respectively). After incubation (3 h, 37°C), the cells were washed twice in PBS and harvested by cell scraping. Cell membranes were isolated with Compartmental Protein Extraction Kit (Chemicon International, Temecula CA) according to the manufacturer's instructions. Yields were typically 60–100 μg of membrane proteins per flask of which 10 μg were used for western blot analysis.

### Statistical analysis

Comparisons between groups were performed by Student's two-tailed t-test. Values are reported as mean (± SD).

## Authors' contributions

ØLH prepared conditioned media and performed affinity chromatography and the accompanying western blot and flow cytometric analyses. He also performed gel filtration and the accompanying western blot and flow cytometric analyses. He performed incubations of membrane fractions with conditioned media and the accompanying western blot analyses, and also performed incubations with ammonium chloride and nocodazole and the accompanying western blot analyses, as well as all experiments involving hypertonic media. JC incubated EC-LDLR-His with conditioned media and performed the accompanying western blot analyses. KEB designed and made the EC-*LDLR*-His and WT-*PCSK9*-RED constructs. TR designed and made the *PCSK9*-His constructs, performed membrane isolations and participated in the gel filtration experiments. TPL conceived the idea of the study, drafted the manuscript and wrote the final version. All authors participated in the design of the study, took part in the writing process and approved the final manuscript.
